# Anosmia and dysgeusia in COVID-19: A systematic review

**DOI:** 10.12688/wellcomeopenres.15917.1

**Published:** 2020-05-13

**Authors:** Rodrigo M. Carrillo-Larco, Carlos Altez-Fernandez

**Affiliations:** 1 Department of Epidemiology and Biostatistics, School of Public Health, Imperial College London, London, SW7 2AZ, UK; 2 CRONICAS Centre of Excellence in Chronic Diseases, Universidad Peruana Cayetano Heredia, Lima, Peru; 3 Instituto de Investigación, Universidad Católica de Trujillo, Chimbote, Peru; 4 Facultad de Medicina Alberto Hurtado, Universidad Peruana Cayetano Heredia, Lima, Peru

**Keywords:** COVID-19, smell disorders, taste disorders, neurological symptoms

## Abstract

**Background: **This systematic review had three aims: i) to determine the frequency of anosmia (or other smell disorders) and dysgeusia (or other taste disorders) in COVID-19 patients; ii) to determine whether anosmia or dysgeusia are independently associated with COVID-19 diagnosis; and iii) to determine whether anosmia or dysgeusia are prognostic factors for impaired outcomes among COVID-19 patients.

**Methods:** On April 20 ^th^, 2020, we search MEDLINE, Embase, Global Health, Scopus, Web of Science and MedXriv. We used terms related to COVID-19, smell and taste disorders. We selected case series, cross-sectional, case-control and cohort studies. We included studies with COVID-19 patients describing their symptoms; studies that compared smell and taste disorders between COVID-19 patients and otherwise healthy subjects; and studies comparing smell and taste disorders between COVID-19 severe and mild/moderate cases. Because of methodological heterogeneity and the limited number of results, a qualitative synthesis is presented.

**Results: **From 31 reports, we selected six (n=2,757). Six studies reported the proportion of smell and taste disorders among COVID-19 patients. Two reports studied whether smell and taste disorders were independently associated with COVID-19 diagnosis. No reports studied the association with impaired outcomes among COVID-19 patients. The frequency of anosmia ranged between 22%-68%. The definition of taste disorders varied greatly, with dysgeusia present in 33% and ageusia in 20%. People who reported loss of smell and taste had six-fold higher odds of being COVID-19 positive; similarly, anosmia and ageusia were associated with 10-fold higher odds of COVID-19 diagnosis.

**Conclusions:** The frequency of smell and taste disorders is as high as other symptoms, thus, at least anosmia for which the definition was more consistent, could be included in lists of COVID-19 symptoms. Although there is promising evidence, it is premature to conclude that smell and taste disorders are strongly associated with COVID-19 diagnosis.

**Registration:** PROSPERO CRD42020181308

## Introduction

COVID-19 is certainly the greatest global health problem nowadays and for the foreseeable future. Clinicians and scientists from all over the world have been producing evidence to understand the epidemiology, clinical profile and prognostic factors of COVID-19. Ever since the first report was published, the world has moved from knowing a few symptoms and risk factors to a large list of COVID-19 symptoms that can also be used for screening and risk stratification purposes ^1^. However, as more evidence becomes available, it is relevant to ascertain its quality and build on this evidence to reach strong conclusions to advance clinical medicine and public health.

Smell and taste disorders, such as anosmia (smell loss) and dysgeusia (taste impairment), have garnered recent attention as potential frequent symptoms and relevant variables for COVID-19 screening ^2– 7^. They are particularly relevant because their assessment does not require interventions or procedures, making them a friendly variable to include in questionnaires or screening algorithms. However, the strength of the evidence for an association between smell and taste disorders and COVID-19 is limited to case reports or anecdotical experiences. Consequently, it is largely unknown whether smell and taste disorders are a frequent symptom among COVID-19 patients, whether they are associated with higher odds of COVID-19 diagnosis, and whether they are prognostic factors for COVID-19 impaired endpoints. To answer these questions and to strengthen the evidence about smell and taste disorders in COVID-19 diagnosis and prognosis, we conducted a systematic review.

## Methods

### Protocol

This systematic review of the scientific literature pursued three aims: i) to determine the frequency of anosmia (or other smell disorders) and dysgeusia (or other taste disorders) in COVID-19 patients; ii) to determine whether anosmia or dysgeusia are independent risk factors for COVID-19 diagnosis; and iii) to determine whether anosmia or dysgeusia are independent prognostic factors for impaired outcomes among COVID-19 patients. We followed the PRISMA reporting guidelines ^8^ (see *Reporting guidelines*) ^9^ and the protocol was prospectively registered at PROSPERO ( CRD42020181308).

### Eligibility criteria

Selected reports included COVID-19 patients (as defined by the original report), men and women. For aims two and three the exposures were anosmia or dysgeusia, both as defined by the original report; similarly, for aims two and three, the comparator was individuals without anosmia or dysgeusia. For the second aim the outcome was COVID-19 diagnosis, whereas for the third aim the outcome was unfavourable endpoints (e.g., admission to intensive care) among COVID-19 patients. For all aims, patients could have been recruited from hospitals or from the community.

We selected observational studies, including case-series, cross-sectional, case-control and retrospective/prospective cohorts. We excluded case reports and trial or intervention studies. We included both published and unpublished materials (pre-prints) and had no language restriction.

### Information sources and search

We used five sources for published materials: MEDLINE, Embase, Global Health, Scopus and Web of Science; the first three through OVID with restriction to reports published in 2020. We also searched MedXriv for unpublished materials. The search was conducted on April 20 ^th^, 2020. The search terms we used are available in Supplementary material table 2 (see *Extended data*) ^9^.

### Study selection

The search results were downloaded to remove duplicate registries. The titles and abstracts were screened to verify whether they met the inclusion criteria described above by two reviewers independently (RMC-L and CA-F). We then studied in detail the reports the two reviewers agreed should be included, as well as those on which the reviewers disagreed. The in-depth evaluation was conducted by two reviewers independently (RMC-L and CA-F); discrepancies at this stage were solved by consensus.

### Data collection

The authors designed a data extraction form and piloted it with two of the selected reports. The form was updated, and one reviewer extracted the information (CA-F) and another verified the extraction (RMC-L); discrepancies were solved by consensus. The information collected included: country where the study took place, sample size, proportion of men and mean age, proportion of anosmia and dysgeusia symptoms (or other smell and taste disorders), and association metrics between exposure and outcomes of interest.

### Risk of bias of individual studies

We used three tools to assess risk of bias, depending on the study design. Studies that assessed the association between anosmia and dysgeusia with COVID-19 were scrutinized with the QUIPS tool ^10^. Conversely, cross-sectional studies were assessed with the Appraisal tool for Cross-Sectional Studies (AXIS) ^11^, and case series were analysed with the IHE Quality Appraisal Checklist for Case Series Studies instrument ^12^. Risk of bias was conducted by one reviewer (CA-F).

### Synthesis of results

We summarized the results qualitatively, by reporting characteristics of the selected reports and studied populations; this included the proportion of COVID-19 patients with anosmia and dysgeusia (or other smell and taste disorders). In addition, we described the association estimates between anosmia and dysgeusia and COVID-19 diagnosis. Because of great heterogeneity in the methods studies used to collect information and the methods used to ascertain anosmia and dysgeusia, we did not pool the proportion of COVID-19 patients with these complaints. Similarly, we did not pool the association estimates because of the limited number of reports.

### Ethics

This is a systematic review of the scientific literature. No human subject participated in the investigation. We did not request approval by an Institutional Review Board or Ethics Committee.

## Results

### Study selection

**Figure 1:**
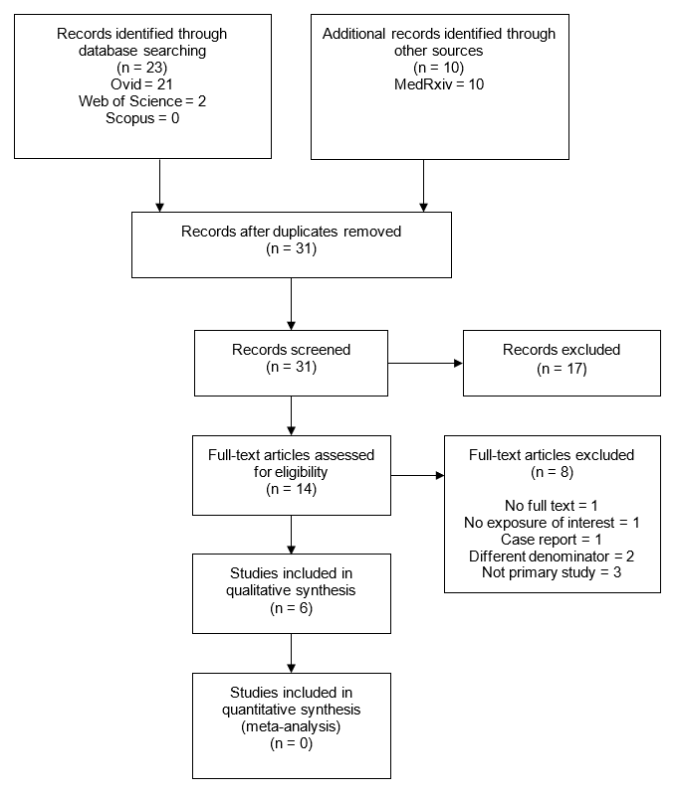
Selection process.

The search yielded 31 results, of which 14 met the inclusion criteria and were studied in detail; finally, six (n=2,757) reports were selected for data extraction ( Figure 1). Of the selected reports ^13– 18^, six provided information for the first aim (frequency of anosmia and dysgeusia in COVID-19 patients) ^13– 18^, and two informed the second aim (association between anosmia and dysgeusia with COVID-19 diagnosis) ^16, 18^. We did not find any reports that studied the association between anosmia and dysgeusia with impaired outcomes (e.g., admission to intensive care) in COVID-19 patients.

### Study characteristics

The studies were conducted in China (n=214) ^15^, Iran (n=120) ^17^, Israel (n=42) ^14^, the UK (n=1,702) ^16^ and US (n=262) ^18^; Lechien and colleagues (n=417) studied patients in four countries in Europe ( Table 1) ^13^. All but one study (case-series) ^15^ followed a cross-sectional design ( Table 1). The mean age of the study participants ranged from 34 to 52 years ^14, 15^; the proportion of men ranged from 28% to 66% ( Table 1) ^16, 17^.

**Table 1.** Anosmia and dysgeusia in COVID-19 patients.

First author	Country	Study design	Total sample	Mean age	Men proportion	COVID-19 proportion	Method of smell problems evaluation	Proportion of smell problems in COVID-19	Method of taste problems evaluation	Proportion of taste problems in COVID+
*Lechein*	Belgium; France; Spain; Italy	Cross-sectional	417	36.9	36.9	1.00	Consultation or online questionnaire for house-bound patients	Anosmia = 68.1% (284/417); Hyposmia = 17.5% (73/417)	Consultation or online questionnaire for house- bound patients	Reduced/ discontinued taste = 78.9%; Distorted taste = 21.1%
*Moein*	Iran	Cross-sectional study in which COVID-19 cases were 1:1 matched with people of a previous study	120	46.5 (among COVID-19 patients)	66.6 (among COVID-19 patients)	0.5	University of Pennsylvania Smell identification Test (UPSIT) assisted by a trained examiner	Normosmia = 2% (1/60); Mild microsmia = 13% (8/60); Moderate microsmia = 27% (16/60); Severe microsmia = 33% (20/60); Anosmia = 25% (15/60)	Not reported	Not reported
*Yan*	USA	Cross-sectional	262	17.6% (60+ years)	37.4	22.5	Questionnaire through email	Anosmia = 22% (13/59)	Questionnaire through email	Ageusia= 20.3% (12/59)
*Levinson*	Israel	Cross-sectional	42	34.0	54.8	1.00	Questionnaire through mobile phone or email	Anosmia= 35.7% (15/42)	Questionnaire through mobile phone or email	Dysgeusia= 33.3% (14/42)
*Mao*	China	Case series	214	52.7	40.7	1.00	Electronic medical records based on the evaluation of two neurologists	Smell Impairment= 5.1% (11/214)	Electronic medical records based on the evaluation of two neurologists	Taste impairment= 5.6% (12/214)
*Menni*	UK	Cross-sectional	1702	41.1	28.0	34.0	Questionnaire through mobile app	Loss of taste and smell = 59.4% (343/579)	Questionnaire through mobile app	Loss of taste and smell = 59.4% (343/579)

There was great heterogeneity regarding how the information was collected. Most researchers used questionnaires, and these were applied through email, apps and mobiles ( Table 1). Moein *et al.* used a validated questionnaire (University of Pennsylvania Smell identification Test) ^17^, while Mao’s group used electronic medical records based on information collected by neurologists ( Table 1) ^15^. There was also a lack of detail on how anosmia and dysgeusia were ascertained, and some authors used broader categories like ‘smell impairment’ or ‘loss of taste and smell’ ( Table 1).

### Frequency of anosmia and dysgeusia among COVID-19 patients

The frequency of anosmia in COVID-19 patients ranged from 22% to 68% ^13, 18^. The definition of taste impairment was more heterogenous, with dysgeusia present in 33% of COVID-19 patients ^14^, ageusia in 20% ^18^, and distorted taste was found in 21% of patients with COVID-19 ^13^.

### Association between anosmia and dysgeusia with COVID-19

Two reports studied the association between anosmia and dysgeusia with COVID-19 diagnosis ^16, 18^, and we did not find any reports studying anosmia and dysgeusia with impaired outcomes in COVID-19 patients.

Menni and colleagues found that people with loss of smell and taste had six-fold higher odds of being COVID-19 positive ( Table 2) ^16^. Similarly, Yan *et al.* found that people presenting anosmia had 10-fold higher odds of being diagnosed with COVID-19 ^18^ ( Table 2). Yan’s work also studied taste disorders and reported that people with ageusia had 10-fold higher odds of having COVID-19 ^18^ ( Table 2). Notably, all the association estimates were adjusted for co-variates including age, sex and other symptoms ( Table 2) ^16, 18^.

**Table 2.** Association between anosmia and dysgeusia with COVID-19 diagnosis.

First author	Exposure	Method of exposure evaluation	Outcome	Outcome definition	Exposure proportion in COVID+	OR for COVID-19 diagnosis (95% CI)	Adjusted for
*Menni*	Loss of taste and smell	Questionnaire through app	COVID-19 infection	rt-PCR confirmation	59.41%	6.59 (5.25-8.27)	Sex, age, BMI
*Yan*	Anosmia	Questionnaire through email	COVID-19 infection	rt-PCR confirmation	22.00%	10.92 (5.08-23.53)	Myalgia/Arthralgia; fatigue; fever; nausea; sore throat
*Yan*	Ageusia	Questionnaire through email	COVID-19 infection	rt-PCR confirmation	20.30%	10.23 (4.74-22.09)	Myalgia/Arthralgia; fatigue; fever; nausea; sore throat

OR, odds ratio; PCR, polymerase chain reaction; BMI, body mass index.

### Risk of bias

The reports by Menni *et al.* and Yan *et al.* were assessed with the QUIPS tool. These studies showed low risk of bias in three criteria: study participation, outcome measurement and statistical analysis and reporting; similarly, they both had moderate risk of bias in the criteria: prognostic factor measurement and study confounding. These studies were assessed differently regarding the study attrition criterion: Menni’s work had low risk whereas Yan’s study showed moderate risk of bias ( Table 3). Overall, the risk of bias assessment for the other studies did not reveal an alarming high risk of bias (see *Extended data*) ^9^.

**Table 3.** Risk of bias of independent studies.

	Study participation	Study attrition	Prognostic factor measurement	Outcome measurement	Study confounding	Statistical analysis and reporting
*Menni*	Low risk	Low risk	Moderate risk	Low risk	Moderate risk	Low risk
*Yan*	Low risk	Moderate risk	Moderate risk	Low risk	Moderate risk	Low risk

## Discussion

### Main findings

This systematic review and critical appraisal of the scientific evidence showed that anosmia may be present in one of every five COVID-19 patents; on the other hand, the frequency of taste disorders varied greatly depending on the definition. This review also found two studies that assessed the association between smell and taste disorders with COVID-19 diagnosis; both studies showed a strong association between anosmia and COVID-19 diagnosis, as well as between ageusia and COVID-19 diagnosis. Notably, these associations were adjusted for socio-demographic variables and other symptoms. There were no reports that studied the association between smell and taste disorders with impaired endpoints among COVID-19 patients.

### Implications for clinical practice

Available evidence may suggest that anosmia is frequently found among COVID-19 patients, as much as or even more frequently than other symptoms ^19– 22^. This may be the reason why the American Centres for Diseases Control and Prevention included smell loss in their list of COVID-19 symptoms ^23^; however, smell loss was not included in the list by the World Health Organization ^24^. Our results may support including anosmia in the lists of COVID-19 symptoms.

The American Centres for Diseases Control and Prevention included taste loss in their list of COVID-19 symptoms ^23^, yet the World Health Organization did not ^24^. Our review found huge heterogeneity on how dysgeusia was assessed and defined. Therefore, our results do not support including taste impairment as a COVID-19 symptom. As more evidence becomes available, ideally including a large sample of patients and consistent definitions, this recommendation can be revisited.

It has been argued that smell loss could be useful for COVID-19 screening ^25, 26^, and it certainly has a huge advantage as its assessment may be inexpensive and harmless ^27^. Our review found two reports signalling that anosmia or smell impairment was independently associated with COVID-19 diagnosis. Nonetheless, and despite the promising evidence ^16, 18^, it seems premature to undoubtedly conclude that anosmia is a strong risk factor for COVID-19 diagnosis or that it can be a successful screening test.

### Implications for research

The clinical and research community will keep on producing evidence about COVID-19, and the associated risk and prognostic factors. Nevertheless, studies with consistent methodologies and definitions are much needed to make comparisons and reach strong conclusions. We invite neurology and otorhinolaryngology professionals to propose a standard definition for anosmia and dysgeusia, along with recommendations on how to assess these symptoms. This way, and as more evidence is published, we will have a better understanding of the role of anosmia and dysgeusia in COVID-19 diagnosis and prognosis.

### Limitations of the review

We conducted a systematic review using six data sources, including one for unpublished materials. We followed standard methods and used relevant tools to appraise the risk of bias. Nonetheless, there are a few limitations we need to acknowledge. The search did not offer any results from Latin America or Africa. Probably, reports from these regions are in local journals not included in the five search engines we used, or it takes a longer time to have their reports uploaded. Future studies in these regions could include regional search engines or other local sources of grey literature. We did not adhere to a strict definition of anosmia or dysgeusia, trying to retrieve as much evidence as possible; despite this decision, the search did not give many results.

### Limitations of the selected reports

The selected reports provided relevant information, though there was great heterogeneity regarding how the exposure of interest was defined and ascertained, and how the information was collected. Also, they studied a limited number of patients. There seems to be a dearth of tools to assess anosmia and dysgeusia, yet Moein and colleagues used the University of Pennsylvania Smell identification Test ^17^. This review provides a comprehensive framework of available evidence about anosmia and dysgeusia in COVID-19, so that researchers interested in this field can build on and advance the available evidence. This could include using standard definitions for the exposure variables and including more patients, ideally from multiple sites or countries, like the work by Lechein and colleagues ^13^.

### Conclusions

Although anosmia seems to be a frequent finding among COVID-19 patients, and it was independently associated with COVID-19 diagnosis, the evidence is still insufficient to claim that anosmia is a strong predictor for COVID-19 diagnosis. The evidence for dysgeusia is much more limited. As the clinical and research community struggle to find predictors to early identify COVID-19 cases, several potential variables should be considered yet studied thoroughly before they can be recommended as risk factors or to be included in risk stratification tools.

## Data availability

### Underlying data

All data underlying the results are available as part of the article and no additional source data are required.

### Extended data

Figshare: Systematic Review: Anosmia/Dysgeusia & COVID-19. https://doi.org/10.6084/m9.figshare.12203435.v2 ^9^

The Supplementary Material DOCX file contains the following extended data:

- Supplementary material table 2 (search terms)
- Risk of bias for Lechien *et al.*, Levinston *et al.*, Moein *et al.*, and Mao *et al.*

### Reporting guidelines

PRISMA checklist for ‘Systematic Review: Anosmia/Dysgeusia & COVID-19’. https://doi.org/10.6084/m9.figshare.12203435.v2 ^9^
