# Supplementary material for: Anosmia and dysgeusia in COVID-19: A systematic review
Source: Wellcome Open Res. 2020 May 13;5:94. [Version 1] doi: 10.12688/wellcomeopenres.15917.1 (PMC7308993; doi:10.12688/wellcomeopenres.15917.1)
Supplement: Peer review history

# Peer review: Reviewer response for version 1

**DOI:** 10.21956/wellcomeopenres.17459.r38974

**Published:** 2020-06-17

**Authors:** Marlene M. Speth

**Affiliations:** 1 Klinik für Hals-, Nasen-, Ohren- Krankheiten, Hals-und Gesichtschirurgie, Kantonsspital Aarau, Aarau, Switzerland

In this paper, the authors perform a systematic review on anosmia and dysgeusia in COVID-19 patients and define three aims: to determine 1) the prevalence of anosmia and dysgeusia, 2) whether there are independent associations of anosmia or dysgeusia with the disease and 3) whether those symptoms influence the course of the disease.

**1. **My major concern with this study is a concern that the authors have expressed as well: that the various studies ascertaining prevalence of olfactory and taste dysfunction in COVID-19, and which have been included, are quite heterogeneous in design/methods (specifically asking patients about olfactory and taste dysfunction vs. chart review) and patient populations (e.g. mild COVID-19, those with flu-like symptoms, non-hospitalized vs. hospitalized). The degree of heterogeneity is too much to synthesize the data and the authors have correctly not performed a meta-analysis. However, I also question whether these data - especially in light of the continually increasing data that are being published on this topic - are sufficient for a systematic review.

**2. Title:** “Anosmia and dysgeusia in COVID-19…”

- A) The authors also mention hyposmia, mild microsmia, smell impairment. Why would therefore the title not be “Olfactory dysfunction and dysgeusia in COVID-19…”

**3. Introduction:**

- A) Further, the authors state that smell and taste disorders are particularly relevant because their assessment does not require interventions or procedures, making them a “friendly” variable to include in questionnaires or screening algorithms.
- This might be true, I believe that the symptoms of smell and taste disorders as symptoms of COVID disease are even more important, as many COVID positive patients experience smell and taste disorders as one of their symptoms and sometimes also as their only symptom. Therefore in my opinion it is not the potential “easiness” of assessment/includement in a questionnaire/history of why smell and taste disorders are so important regarding the disease, but more so as positive patients who “only” suffer from smell and/or taste disorders might be possible spreaders of the disease and therefore it is important to render attention to those patients.
- B) Further in the introduction it is stated that it is largely unknown whether smell and taste disorders are a frequent symptom among COVID-19 patients. I believe many recent articles have shown that smell and taste disorders are frequent symptoms of COVID-19 patients (see for example Lechien *et al.*, 2020 ^1^).
- C) In additions the authors state that it is further unknown whether smell and taste disorders are associated with higher odds of COVID-19 diagnosis, and whether they are prognostic factors for COVID-19 impaired endpoints. Please specify that you mean **new onset** smell and taste disorders.

**4. Methods:**

 A) Eligibility criteria: “the comparator was individuals without anosmia or dysgeusia”.

- grammar: “ the comparator were individuals..:”

B) I disagree with using articles from a preprint server. At this point, there are so many articles that have been published on olfactory dysfunction and taste dysfunction in COVID-19, that we should be relying entirely on peer-reviewed studies.

**5. Results:**

- A) Study characteristics: “Moein *et al.* used a validated questionnaire (UPSIT)”. The UPSIT is an objective smell test, thus the word “questionnaire” sounds a bit confusing.
- B) Frequency of anosmia and dysgeusia among COVID-19 patients: “The definition of taste impairment was more heterogeneous, with dysgeusia present [..], ageusia in […] , and distorted taste in […].
- C) I do not understand why the “definition of taste impairment was more heterogeneous? Dysgeusia is something different than ageusia.
- D) Association between anosmia and dysgeusia with COVID-19: As stated above, please specify that you are referring to **new onset** anosmia and dysgeusia.
- E) Table 2: Exposure: to be coherent, I would suggest to either write about loss of taste and smell or about anosmia or ageusia.

**6. Discussion:**

- A) Main findings: “This systematic review […] in one of every five COVID-19 patents.” - a grammar/typo: “patents”, please change to “patients”.
- B) Implications for clinical practice: “Our results may support including anosmia in the list of COVID-19 symptoms.” In the paper, the authors also included hyposmia, what about this, would it be included as a symptom and what does “may support including” mean? Did the authors come to the conclusion that **olfactory dysfunction** should be included as a symptom or not or were they not able to draw a conclusion based on what factors?
- C) The authors further come to the conclusion that they do not support including taste impairment as a COVID-19 symptom due to the huge heterogeneity on how dysgeusia is assessed and defined. However, what the authors of the paper cite as heterogeneity in their paper is partly also different medical terms for different medical issues. Thus, it would be utterly important for the authors to study and use the correct medical term.

**7. Implications for research:**

- “Nevertheless studies with consistent methodologies and definitions are much needed to make comparisons and reach strong conclusions. We invite neurology and otorhinology professionals to propose a standard definition for anosmia and dysgeusia, along with recommendations on how to assess these symptoms”.
- Anosmia and dysgeusia are medical terms, which have already been defined (see for example, the Position Paper on Olfactory Dysfunction by Hummel *et al.*, 2017 ^2^). Recommendations on how to assess these symptoms have also been made in the past.
- It is of importance to also use these medical terms correctly. Anosmia, dysosmia, hyposmia, ageusia, dysgeusia are in fact not terms applied “heterogeneous”, but are terms used for talking about different medical conditions.

Are the rationale for, and objectives of, the Systematic Review clearly stated?

Yes

Is the statistical analysis and its interpretation appropriate?

Partly

Are sufficient details of the methods and analysis provided to allow replication by others?

Yes

Are the conclusions drawn adequately supported by the results presented in the review?

Partly

Reviewer Expertise:

Rhinology, chronic rhinosinusitis, allergy, olfactory disfunction in covid patients

I confirm that I have read this submission and believe that I have an appropriate level of expertise to state that I do not consider it to be of an acceptable scientific standard, for reasons outlined above.

## References

[1] : Olfactory and gustatory dysfunctions as a clinical presentation of mild-to-moderate forms of the coronavirus disease (COVID-19): a multicenter European study. *Eur Arch Otorhinolaryngol*.2020; 10.1007/s00405-020-05965-1 10.1007/s00405-020-05965-1 32253535PMC7134551

[2] : Position paper on olfactory dysfunction. *Rhinol Suppl*.54(26) : 10.4193/Rhino16.248 1-30 10.4193/Rhino16.248 29528615

# Peer review: Reviewer response for version 1

**DOI:** 10.21956/wellcomeopenres.17459.r38977

**Published:** 2020-06-16

**Authors:** B. Nirmal Kumar, John Rocke

**Affiliations:** 1 Wrightington, Wigan and Leigh NHS Foundation Trust, Wigan, UK

Thanks for the invitation to perform open peer review. This is a very important topic of great public health importance as appropriate measures can reduce the spread of the virus and help contain the pandemic. 

- Are the rationale for, and objectives of, the Systematic Review clearly stated?

The three aims for the systematic review are clearly stated in the context of the COVID-19 outbreak. Identifying the frequency of these symptoms and risk factors for diagnosis is possible in the early stages of this pandemic but the 3rd aim of determining prognostic factors for outcomes is a little unrealistic considering the timeline. 

 There is clearly interest in the utility of anosmia and dysguesia and this is a current and appropriately framed review.

 The review does not state that the World Health Organisation has now recognized loss of smell as a key symptom in COVID-19 as the review was published in April.

  

- Are sufficient details of the methods and analysis provided to allow replication by others?

Search terms are included in the Appendix for future replication of searches. Prisma diagram demonstrates review process accurately. The review is framed towards anosmia but search terms also include “smell dysfunction” and “smell impairment” which are terms that are not truly anosmia. The authors may want to consider using another term instead of anosmia to better reflect their search strategy. This will help match the dysguesia search term more closely as one of their aims is to assess if each are independently associated with diagnosis.

 They selected only 6/31 reports and several notable studies have not been included in this review including Giacomelli *et al.*, Kaye *et al*. and Bagheri *et al.* Some of these articles may have been excluded due to the timing of this review but perhaps selection criteria for inclusion was limited.

  

- Is the statistical analysis and its interpretation appropriate?

No statistical analysis was undertaken in this study.

  

- Are the conclusions drawn adequately supported by the results presented in the review?

Reasonable and sensible conservative conclusions are drawn. Whilst olfactory dysfunction and anosmia may not be a strong predictor it seems, from the studies included, that it may be a better predictor than cough, fever or dyspnoea. As such it is important that this is recognised by the authors in their conclusions so that the symptom is recognised by public health bodies internationally.

Are the rationale for, and objectives of, the Systematic Review clearly stated?

Yes

Is the statistical analysis and its interpretation appropriate?

Not applicable

Are sufficient details of the methods and analysis provided to allow replication by others?

Yes

Are the conclusions drawn adequately supported by the results presented in the review?

Yes

Reviewer Expertise:

NA

We confirm that we have read this submission and believe that we have an appropriate level of expertise to confirm that it is of an acceptable scientific standard.

# Peer review: Reviewer response for version 1

**DOI:** 10.21956/wellcomeopenres.17459.r38756

**Published:** 2020-05-19

**Authors:** Luigi Angelo Vaira

**Affiliations:** 1 Maxillofacial Surgery Operative Unit, University Hospital of Sassari, Sassari, Italy

Thanks for the opportunity to review this manuscript.

 First, I would like to commend the authors for having collected the data currently present in the literature, regarding olfactory dysfunctions in COVID-19 patients.

 The review proposed by the authors provides a useful summary of findings reported until 20th April. The review methodology is clearly stated and described and the data analysis and interpretation is correct.

 I have only two minor comments:

1. At the beginning of the discussion section, the authors stated "there were no reports that studied the association between smell and taste disorders with impaired endopoints among COVID-19 patients". I believe that Moein *et al.* gives some prognostic indications without detecting significant correlations between the severity of chemosensitive disorders and the severity of COVID-19.
2. The topic is rapidly evolving and, after April 20, some studies have been published based on objective assessments with psychophysical tests. Before April 20, as rightly reported by the authors, the only one present in the literature was that of Moein *et al.* These studies have shown that observational or interviewing studies (such as almost all the studies published before April 20) underestimate the frequency of chemosensitive disorders. The latter studies show a high recall bias as many patients, who at the time of the visit no longer present the dysfunction, cannot remember it, often distracted by the seriousness of the general clinical picture. At the same time, only objective studies can allow to accurately quantify the disorder and differentiate it in the various clinical subcategories (e.g. anosmia, severe, moderate, mild hyposmia). Something in this sense could be added to the limitations of the selected reports in this sense.

Are the rationale for, and objectives of, the Systematic Review clearly stated?

Yes

Is the statistical analysis and its interpretation appropriate?

Yes

Are sufficient details of the methods and analysis provided to allow replication by others?

Yes

Are the conclusions drawn adequately supported by the results presented in the review?

Yes

Reviewer Expertise:

Maxillofacial surgery

I confirm that I have read this submission and believe that I have an appropriate level of expertise to confirm that it is of an acceptable scientific standard.
